# A Wearable Head Mounted Display Bio-Signals Pad System for Emotion Recognition

**DOI:** 10.3390/s22010142

**Published:** 2021-12-26

**Authors:** Chunting Wan, Dongyi Chen, Zhiqi Huang, Xi Luo

**Affiliations:** 1 School of Automation Engineering, University of Electronic Science and Technology of China, Chengdu 611731, China; wanchunting@std.uestc.edu.cn (C.W.); zhiqih@uestc.edu.cn (Z.H.); 201921060623@std.uestc.edu.cn (X.L.); 2 School of Electronic Engineering and Automation, Guilin University of Electronic Science and Technology, Guilin 541004, China

**Keywords:** emotion recognition, bio-signals, Head-Mounted Displays, virtual reality, HCI interface

## Abstract

Multimodal bio-signals acquisition based on wearable devices and using virtual reality (VR) as stimulus source are promising techniques in emotion recognition research field. Numerous studies have shown that emotional states can be better evoked through Immersive Virtual Environments (IVE). The main goal of this paper is to provide researchers with a system for emotion recognition in VR environments. In this paper, we present a wearable forehead bio-signals acquisition pad which is attached to Head-Mounted Displays (HMD), termed HMD Bio Pad. This system can simultaneously record emotion-related two-channel electroencephalography (EEG), one-channel electrodermal activity (EDA), photoplethysmograph (PPG) and skin temperature (SKT) signals. In addition, we develop a human-computer interaction (HCI) interface which researchers can carry out emotion recognition research using VR HMD as stimulus presentation device. To evaluate the performance of the proposed system, we conducted different experiments to validate the multimodal bio-signals quality, respectively. To validate EEG signal, we have assessed the performance in terms of EEG eyes-blink task and eyes-open and eyes-closed task. The EEG eyes-blink task indicates that the proposed system can achieve comparable EEG signal quality in comparison to the dedicated bio-signals measuring device. The eyes-open and eyes-closed task proves that the proposed system can efficiently record alpha rhythm. Then we used signal-to-noise ratio (SNR) and Skin Conductance Reaction (SCR) signal to validate the performance for EDA acquisition system. A filtered EDA signal, with a high mean SNR of 28.52 dB, is plotted on HCI interface. Moreover, the SCR signal related to stimulus response can be correctly extracted from EDA signal. The SKT acquisition system has been validated effectively by the temperature change experiment when subjects are in unpleasant emotion. The pulse rate (PR) estimated from PPG signal achieved the low mean average absolute error (AAE), which is 1.12 beats per minute (BPM) over 8 recordings. In summary, the proposed HMD Bio Pad offers a portable, comfortable and easy-to-wear device for recording bio-signals. The proposed system could contribute to emotion recognition research in VR environments.

## 1. Introduction

Automated emotion recognition (AEE) is a process of automatic recognition to human emotional responses by computers. It is an important research branch in the field of human-computer interaction (HCI) [1]. The use of AEE has great potential in various intelligent systems, including education (students’ learning status assessment), marketing (customers’ feedback assessment), and mental health monitoring (patients’ emotional states adjustment) [2]. Emotion recognition can be achieved through facial expression, body posture, voice and bio-signals, etc. [3]. Among them, bio-signals can further objectively and truly express human emotions because of their non-subjective manipulation [1,4].

In recent years, with the development of virtual reality (VR) technology, emotion recognition based on multimodal bio-signals in VR environments has become a hot research topic [5,6]. VR creates an immersive virtual environment for users and allows them to experience the real feelings [7]. It is very important for the research in the field of psychology, which is also considered as the most likely prospective research technique to replace real and laboratory environments. Up to the present, the music, pictures, videos and other non-immersive stimulating materials are used by most of researchers to evoke emotions. Moreover, it has been proved that VR scenes can evoke the emotion of users better [8].

Bio-signals show excellent data consistency in emotion analysis because they represent an unfiltered immediate response to human emotions. At present, bio-signals used in emotion analysis mainly include electroencephalogram (EEG) [9,10,11], photoplethysmogram (PPG) [12,13,14], electrodermal activity (EDA) [15,16] and skin temperature(SKT) [17,18], etc. There are mainly three types of devices for acquiring bio-signals. One is the special medical devices, such as electroencephalograph, electrocardiograph, electromyography, pulse oximeter and thermometer. However, there is a problem of data synchronization in the combination of multiple devices. The second category is dedicated bio-signals measuring devices, such as Biopac MP150, Procomp Infiniti and Power Lab, etc. The advantage of these dedicated bio-signals measuring devices is that these can collect multiple bio-signals synchronously. The common drawbacks of these two types of devices are high price, cumbersome cables and time-consuming wearing.

With the development of wearable physiological detection, bio-signals sensors can already be built into terminals such as clothes, hats, shoes, gloves, beds and game handles for long-term users’ emotional perception and feedback. The Empatica company launched E4 smart watch to collect data for affective computing researchers [19]. This watch can collect EDA, PPG and SKT at the wrist, and can also calculate pulse rate (PR) through PPG signal. The reference [20] embedded dry electrodes EEG sensor and two eye-tracking cameras in the HTC Vive headset. It can synchronously record muli-channel EEG signals and eye movement image. Moreover, the authors of [20] provided an emotion recognition interface for predicting users’ evaluations of attractiveness. While there are two disadvantages in the system, one is that it cannot acquire other peripheral bio-signals which is vital for emotion recognition. The other is that the EEG sensors module cannot be equipped with other VR or AR HMD on the market. A single dry-electrode wearable device NeuroSky MindWave was applied to collect prefrontal EEG signal to connect personality traits and emotional states in [21]. The wearable devices Emotive EPOC and SHIMMER are used in [22] to collect 14-channel EEG signals and one-channel PPG signal in five VR game scenes, respectively. The results suggested that the bio-signals acquired by low-cost wearable devices could be employed to recognize emotion, with high precision. All the above researches indicate that with the development of wearable technology, the effectiveness of the use for wearable devices to collect bio-signals for emotion recognition has proven. However, there are still some drawbacks in the above mentioned wearable devices for emotion recognition in VR environments. For example, none of the wearable devices mentioned above can synchronously collect emotion-related EEG signals and other peripheral bio-signals. In addition, these devices lack a unified interface to perform stimulus selection, data acquisition, and emotion modeling simultaneously.

In this paper, we propose a wearable forehead bio-signals acquisition platform called HMD Bio Pad which can be attached to most of the VR or AR HMD on the market using a velcro fastener. The HMD Bio Pad hardware consists of flexible sensors pad and bio-signals acquisition system. Flexible sensors pad is designed by flexible printed circuit board (PCB) which can be bent at will. The metal dry electrodes and sensors are placed on the flexible sensors pad. Bio-signals acquisition system is mainly responsible for signal conditioning, acquisition and transmission. Two-channel EEG signals, one-channel EDA signal, PPG signal and SKT are recorded simultaneously via Bluetooth when users ordinarily wear HMD Bio Pad. Wireless communication supports the portability of HMD Bio Pad. Then we conducted different experiments to evaluate the performances of EEG signal, EDA signal, PPG signal and SKT. Finally, we developed a user-friendly HCI interface for the researches in field of psychophysiology in VR environments. The major contributions of this work can be summarized as follows:

- A wearable forehead bio-signals acquisition device called HMD Bio Pad is developed which has the advantages of portability, comfort and ease of wearing;
- Using metal dry electrodes and attaching flexible sensors pad to HMD can greatly reduce experimental preparation time and improve the convenience of the system.
- HMD Bio Pad can simultaneously collect EEG signals and other peripheral bio-signals, of which performances have been validated by different experiments;
- A HCI interface is provided for researchers to perform stimulus selection, data acquisition, and emotion modeling simultaneously.

The remainder of this paper is organized as follows: Section 2 describes the system overview. Section 3 and Section 4 introduce HMD Bio Pad hardware and software platform respectively. Evaluation of HMD Bio Pad is presented in Section 5. Conclusions and future works are discussed in the last Section.

## 2. System Overview

**Figure 1:**
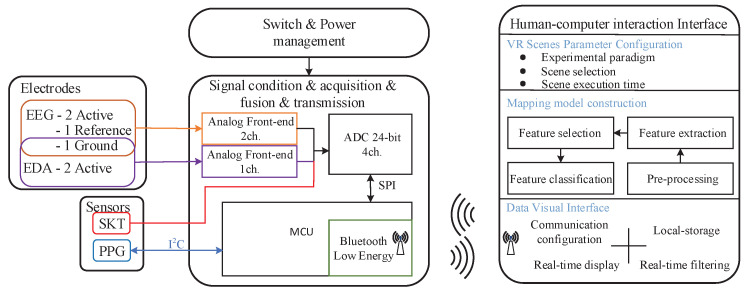
The block diagram of the proposed system.

The block diagram of HMD Bio Pad is shown in Figure 1. The overall system design can be divided into three parts.

(1) Flexible sensor pad: The flexible PCB is composed of golden dry electrode and sensors (PPG, Temperature, Acceleration, etc.), which is connected with bio-signals acquisition system through USB Type-C connector;
(2) Bio-signals acquisition system: The system contains switch and power management block including battery charging and power supply circuit. It also includes signal conditioning analog front-end (AFE), high-speed data acquisition module, data fusion module and bluetooth low energy (BLE) data transmission module;
(3) Human-computer interaction (HCI) interface: The HCI interface consists of data visualization interface, VR scene parameter configuration interface and mapping model construction interface. Data visualization interface is construsted by real-time waveform display, communication configuration and data storage. The scene parameters can be set by VR scene parameter configuration interface based on the requirements for different experiments. Common machine learning methods can be provided by Mapping model construction interface for emotion modeling.

**Figure 2:**
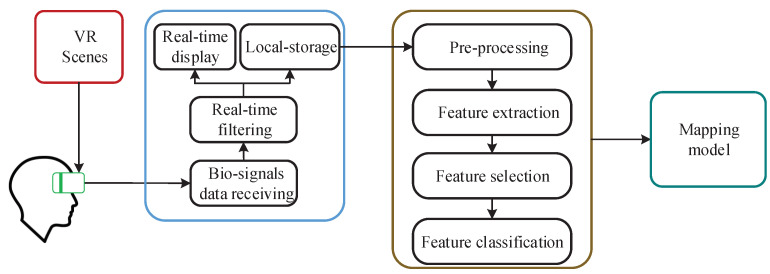
A flow diagram of our proposed system to complete the emotion modeling experiment.

Figure 2 shows a flow diagram of our proposed system to complete the emotion modeling experiment.The VR HMD automatically plays the VR scenes according to the preset experimental paradigm, and the bio-signals of the subjects are recorded simultaneously by HMD Bio Pad. After receiving the recorded bio-signals data on the HCI interface software, the emotion model is constructed through signal pre-processing, feature extraction, feature selection and feature classification.

## 3. The Hardware Design of HMD Bio Pad

**Figure 3:**
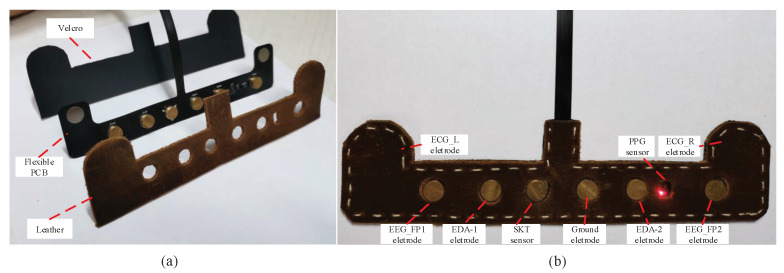
The flexible pad: (**a**) The structure of flexible pad. (**b**) The electrodes and sensors placement on the flexible pad.

In this section, we give the description of the HMD Bio Pad hardware design. The main goal is to design a wearable device which collects multimodal bio-signals from forehead. The HMD Bio Pad hardware consists of flexible sensor pad and bio-signals acquisition system. They are connected by USB Type-C connector. The structure diagram of flexible sensor pad can be seen in Figure 3a. One side of flexible printed circuit board (PCB) is covered with a breathable, skin-friendly leather material, and the other side is covered using a velcro fastener. The electrodes and sensors are placed on the flexible PCB, which are shown in Figure 3b.

Bio-signals acquisition system consists of the following parts: EEG acquisition system, EDA acquisition system, SKT acquisition system, PPG acquisition system, switch, power management, microcontroller unit (MCU) and BLE wireless communication.

### 3.1. EEG Acquisition System

**Figure 4:**
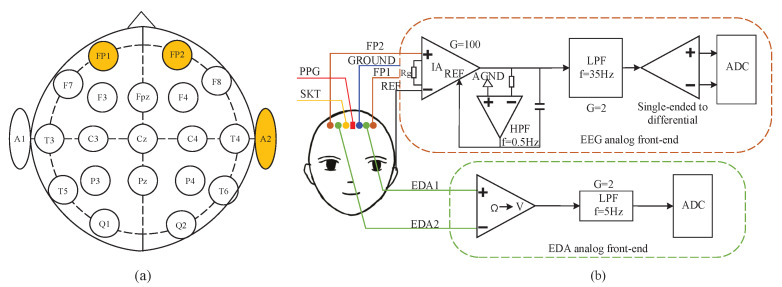
The positions of electrodes and the flowchart of AFE circuit: (**a**) The positions of electrodes placement according to the 10–20 international EEG standards. (**b**) The positions of electrodes and sensors, the AFE circuit of EEG and EDA.

In recent years, numerous neurophysiological researches have been reported the correlations between EEG signals and emotions. Recent studies showed that the frontal scalp seems to store more emotional activation than other regions of brain [23,24]. The EEG asymmetry of the left and right hemispheres is an important reference feature to judge cognitive and affective disorders [25]. Since the forehead is not covered by hair, it has small skin-to-electrode interface impedance, which is more conducive for the use of dry electrodes to collect high-fidelity EEG signal. Therefore, we place dry electrodes on each of the left and right sides of the prefrontal lobe. The reference electrode is applied with a ear-clip which is welded to the bio-signals acquisition system by a shield cable. According to the 10–20 international EEG standards, the FP1 and FP2 are chosen as the positions of active electrodes, the earlobe is used as the position of reference electrode (A2), which form the forehead two-channel EEG signals acquisition system. The electrodes placement positions are shown in Figure 4a.

EEG is a technique for recording the electrophysiological activity of brain neurons on the surface of the cerebral cortex or scalp. The amplitude of EEG in microvolt (μV) order of magnitude is usually very weak. Therefore, EEG signal is susceptible to interference and difficult to be directly detected. It is necessary to design analog front-end (AFE) circuit for signal amplification and conditioning. The two-channel AFE circuit of EEG designed in this paper is shown in Figure 4b.

The AFE circuit of EEG is composed of instrument amplifier (IA) circuit, DC voltage correction circuit, second-order low-pass filter circuit and single-ended to differential circuit. Due to weak amplitude of EEG signal as 10–50 μV, the IA with low input-referred noise, high common-mode rejection ratio (CMRR) and high input impedance was required for the first stage amplification of EEG signal. AD8422 chip (Analog Devices, Norwood, MA, USA) with low input-referred noise (0.1 μVPP), high CMRR (94–150 dB) and high input impedance (200 GΩ) is used as IA. It is the third generation product of AD620 chip (Analog Devices, USA), and the gain *G* is determined by the gain setting terminals resistance $R_{g}$. The range of the gain *G* is 1–1000. It is noted that excessive gain will also amplify the DC voltage contained in EEG and saturate the output of amplifier. Therefore, the gain *G* is set as 100. According to Equation (1), the value of $R_{g}$ is 200Ω.

(1) $$G=1+\frac{19.8\;k\Omega }{R_{g}}$$

According to Equation (2), CMRR is about 120 dB.

(2) CMRR=80dB+20lgG

The transfer function of the AD8422 is given as follows:

(3) $$V_{out}=G*\left(V_{in+}-V_{in-}\right)+V_{ref},$$

where $V_{in+}$ and $V_{in-}$ represent the positive and negative inputs, respectively. $V_{ref}$ denotes the input reference voltage.

The DC voltage introduced by the electrode wires contacting with the scalp is called polarization voltage (millivolt magnitude). The polarization voltage amplified by IA will result in saturation and serious distortion of EEG signal. At the same time, the polarization voltage also limits CMRR of the preamplifier and shortens the gain range of IA. The purpose of the DC voltage correction circuit is to eliminate the polarization voltage. In this paper, the integral feedback circuit with a cut-off frequency of 0.5 Hz is applied to realize DC voltage correction in Figure 4b. After the amplification of the input EEG signal by the IA, the signal passes through the integral feedback circuit. Then the output signal is fed back to the REF pin of IA, the output of the integral feedback circuit can be calculated by Equation (4).

(4) $$V_{ref}=-\frac{1}{RC}*\int V_{out}\;dt$$

We substitute Equation (4) into Equation (3), the output transfer function of the instrument amplifier can be found in Equation (5).

(5) $$V_{out}=G*\left(V_{in+}-V_{in-}\right)-\frac{1}{RC}*\int V_{out}\;dt$$

It can be seen from Equation (5) that the amplified DC offset voltage will be eliminated at the output of the instrument amplifier through the integral circuit. The integral feedback circuit can realize dynamic DC correction.

The EEG signal after IA amplification and DC correction mainly removes the influence of extremely low frequency, while still contains high frequency interference signal in EEG signal. The high frequency interference signal mainly includes environmental electromagnetic waves, electromyography (EMG) signal and noise caused by the active devices. Therefore, it is necessary to design a low-pass filter to eliminate high frequency interference. The frequency range of EEG signal is generally from 0.5 to 100 Hz, but the frequency range of alpha (8–13 Hz), beta (13–30 Hz), theta (4–8 Hz) and delta (0.5–4 Hz) associated with emotion ranges from 0.5 to 30 Hz [2]. In this paper, a second-order low-pass filter with 35-Hz cutoff frequency is designed for eliminating noise from EEG signal. In addition, the filter circuit amplifies 2 times of the EEG signal.

The conversion of single-ended signal into differential signal can effectively reduce the common mode interference and increase the dynamic range of the signal. The output signal of the second-order low-pass filter is converted into a differential signal by performing a single-ended to differential circuit. The differential conversion circuit uses fully differential amplifier THS4521 chip (Texas Instrument, Dallas, TX, USA). THS4521 has very low input noise and power. When the bandwidth is 100 kHz, the voltage noise density is low to 4.6 nV/$\sqrt{\mathrm{Hz}}$, which is very suitable for driving the high precision Σ-Δ type analog-to-digital converter (ADC). The converted differential signal is fed into the high-precision differential ADC, which converts the analog signal into digital signal. The ADC chip ADS1256 (Texas Instrument, Dallas, TX, USA) has extremely low-noise, 24-bit resolution, 4-channel Σ-Δ differential inputs. The ADC conversion result is calculated using the following Equation:

(6) $$ADC_{out}=\left(\frac{2*V_{REF}}{2^{23}-1}*L-5\right)/PGA,$$

where reference voltage $V_{REF}$ = 2.5 V, PGA is the internal gain value, *L* represents the complement of digital quantities collected by the ADC.

### 3.2. EDA Acquisition System

**Figure 5:**
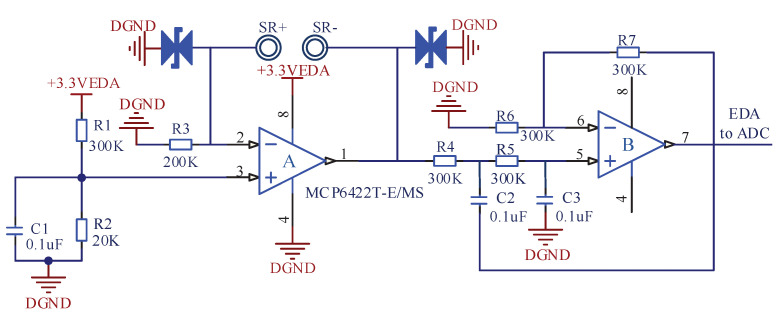
The AFE circuit of EDA.

Electrodermal activity (EDA) refers to the sympathetic response caused by strong emotional stimulation, which leads to the rapid increase of secretion of sweat glands in a short period of time, that is, the generation of mental sweating [26]. Mental sweating is the most obvious on the palmar and plantar sites, and can also be found on the back of the hands, forehead, neck, forearms and legs [27]. In this paper, the EDA electrodes are placed on the forehead, as shown in Figure 4b. The acquisition of EDA converts the change of forehead surface impedance into that of electrical signal. The AFE circuit of EDA is depicted in Figure 5, where SR+ and SR− represent two mental electrodes.

The AFE circuit of EDA consists of the following two stages. The first stage is the Ω/V conversion of forehead surface impedance. Considering the power consumption (4.4 μA/ Amplifier (Typical)), offset voltage (±1 mV (Maximum)), output voltage swing and the number of operation amplifiers, the MCP6422 chip (Microchip Technology, Chandler, AZ, USA) with two internal low-power amplifiers is selected in this paper. According to the principle of “virtual short” and “virtual break” of the operational amplifier, the flowing current from SR+ to SR− is calculated in Ω/V stage. After the calculation, the maximum current going through the human body is less than 10 μA, which is within the safety range of human body. Generally, the impedance of human body range from tens KΩ to hundreds KΩ. Thus, according to the calculation results, the output voltage from the AFE circuit of EDA ranges from 0.4 to 2.4 V. Since the useful frequency range of EDA signal is below 5 Hz [28], the second stage of the circuit uses the classical Sallen-Key second-order active low-pass filter to eliminate the high frequency interference. The cut-off frequency of the low-pass filter can be calculated by Equation (7):

(7) $$f_{p}=\frac{1}{2\pi RC},$$

where R=R4=R5=R6=R7=300KΩ, C=C1=C2=0.1μF. According to the calculation results, the cut-off frequency of the filter is approximately 5.3 Hz. The output EDA signal is calculated by Equation (8):

(8) $$U_{eda}=3.3*\left(\frac{R2}{R1+R2}\right)*\left(1+\frac{R_{eda}}{R3}\right)*\left(1+\frac{R7}{R6}\right),$$

where $R_{eda}$ represents skin impedance. Substituting the marked parameters in Figure 5 into Equation (8), the relationship between $U_{eda}$ and $R_{eda}$ can be obtained as shown in Equation (9).

(9) $$U_{eda}=\frac{3.3}{8}*\left(1+\frac{R_{eda}}{200\;K\Omega }\right)$$

The skin impedance $R_{eda}$ can be obtained by collecting the AFE output $U_{eda}$ through the ADC. EDA signal is generally represented by conductance. According to Equation (9), the solution formula of conductance $\rho_{eda}$ can be obtained as shown in Equation (10):

(10) $$\rho_{eda}=\frac{3.3}{0.2*(8U_{eda}-3.3)})*10^{3},$$

where the unit of $\rho_{eda}$ is μS.

### 3.3. SKT Acquisition System

**Figure 6:**
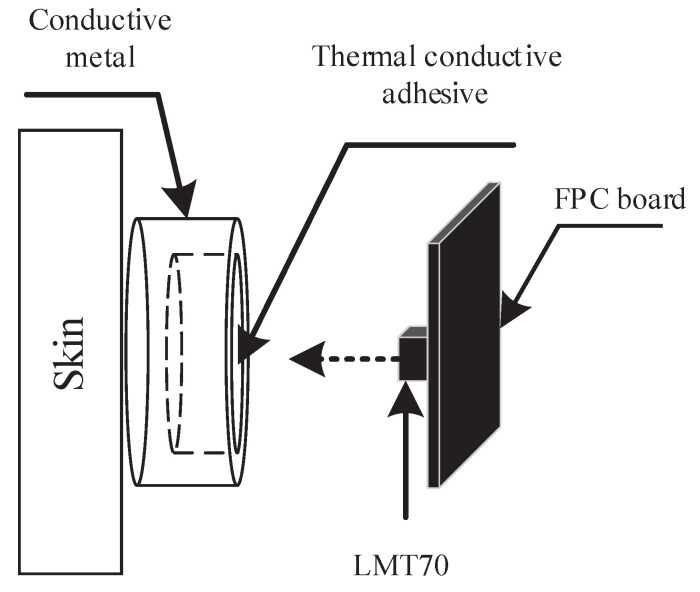
The schematic diagram of the temperature sensor structure.

The change of skin temperature is a manifestation of vascular response. There are slight differences in body skin temperature with different emotional states, which can be used for emotion recognition research [29]. At present, the body temperature measurement sites mainly include oral, rectal, armpit, ear and forehead, etc. In this paper, the integrated temperature sensor is placed at the center of the forehead, as shown in Figure 4b. The integrated contact temperature sensor LMT70 (Texas Instrument, Dallas, TX, USA) with small-size (0.88 mm * 0.8 mm), high-accuracy (20–42 ${}^{{}^{\circ}}C$, ±0.05 ${}^{{}^{\circ}}C$) and low-power consumption (9.2 μA), which is suitable for wearable devices. The schematic diagram of the temperature sensor structure is shown in Figure 6.

LMT70 is welded on the flexible PCB by reflow soldering technology, and the high-performance thermal conductive adhesive is filled into the customized internal hollowing thermal conductive metal. The thermal conductive metal after filling the thermal conductive adhesive is connected and fixed with LMT70. The purpose of this structure design is to keep the conductive metal at the same height with the EEG and EDA metal electrodes, so that the electrodes and the conductive metal can fully contact with forehead skin. Another purpose is to avoid skin injury that may be caused by long-term direct contact of LMT70 with the skin. The output of LMT70 is analog quantity, which needs to be converted into digital quantity through ADC. We use the third channel of ADS1256, and the voltage value $V_{TAO}$ can be calculated according to Equation (6). The temperature value *T* can be calculated by Equation (11):

(11) $$T=m*V_{TAO}+b,$$

where *m* represents slope value, *b* denotes the intercept value. It is known that the forehead temperature range of normal human body is between 30 ${}^{{}^{\circ}}C$ and 40 ${}^{{}^{\circ}}C$ in a laboratory environment. In this paper, according to the corresponding typical values of the voltages associated with 30 ${}^{{}^{\circ}}C$ and 40 ${}^{{}^{\circ}}C$ in the LMT70 chip manual, the slope value *m* and the intercept value *b* are set as 0.1943 ${}^{{}^{\circ}}C$/mv and 213.340 ${}^{{}^{\circ}}C$, respectively.

### 3.4. PPG Acquisition System

The amplitude, period, pulse rate (PR) and pulse rate variability (PRV) of PPG can be used as features of emotion recognition, especially PR. Researches have been reported that when people are in positive emotional state, the corresponding PR value will be low; otherwise, the PR value increases, which is not beneficial to health. The common locations for obtaining PPG signal are fingers, wrists, earlobes and forehead [30]. Studies showed that 4 cm on the left or right of the forehead center is the ideal location for PPG acquisition [31]. In this paper, a reflective PPG sensor is selected and placed on the forehead, as shown in Figure 4b. The PPG sensor selects the high-sensitivity, low-power consumption (<1 mW) MAX30102 chip (Maximum Integrated, San Jose, CA, USA), which integrates 2 internal LEDs (red and infrared LED) and a photodetector. The PPG sensor communicates with the MCU bidirectionally through the $I^{2}C$ bus. MCU can set LED current, sampling frequency, ADC bits and other parameters, and can read the photoelectric detector output results through the $I^{2}C$ bus.

### 3.5. Switch, Power Management, MCU and BLE Wireless Communication

The system can be run or stopped by a long 3 s touch switch. The touch switch-activated green LED is indicates whether the system is running. The power management includes battery charging and power supply circuit. The 3.7 V (full charge around 4.2 V) lithium-ion battery is used for power supply of the whole system. The chip LTC4054 (Linear Technology, Milpitas, CA, USA) is used to control battery charging through the USB Type-c connector. The red charge indicator led extinguish when battery is full. The low dropout regulator(LDO) will generate ±5.0 V, 3.3 V and 2.7 V voltages according to the power supply requirements of the whole system analog and digital circuits. The MCU is mainly responsible for the multimodal physiological data collection, fusion and transmission. Based on the above requirements, this paper selects the low energy Bluetooth SOC nRF52832 (Nordic Semiconductor, Trondheim, NOR). This chip supports Bluetooth 5 protocol and programmable broadcast gain, and its effective data transmission speed is as high as 1447 Kbps. In addition, the overall architecture is based on Arm CortexTM-M4F CPU with built-in floating-point operation unit and DSP processing unit, which can quickly handle complex tasks, so that the CPU can work in a low energy state for a long time. nRF52832 integrates a wealth of digital peripherals, such as: UART, $I^{2}C$ and SPI, etc. The multi-channel Easy DMA and PPI functions allow communication between peripherals without CPU intervention.

**Figure 7:**
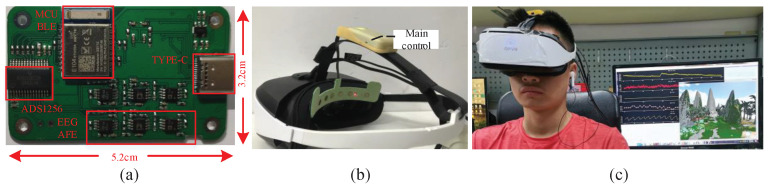
The HMD Bio Pad: (**a**) The PCB of Bio-signals acquisition system. (**b**) Attaching HMD Bio Pad to DPVR E3 VR HMD. (**c**) HMD Bio Pad wearing by a subject.

The PCB of bio-signals acquisition system is shown in Figure 7a. The board size is 5.2 cm × 3.2 cm. The main hardware function modules are marked with red line and tested. An example that HMD Bio Pad is assembled in DPVR E3 VR HMD is depicted in Figure 7b. Figure 7c shows HMD Bio Pad worn by the subject who is in VR scene.

## 4. HCI Interface Software Design

The human-computer interaction (HCI) interface can develop a unified interface for researchers to acquire bio-signals, induce emotions and recognize emotions. It can raise the work efficiency and improve the user experience. The HCI interface is implemented via Python language. The system software is mainly divided into three parts: data visualization interface, experimental paradigm setting interface and emotion modeling interface.

### 4.1. Data Visualization Interface

**Figure 8:**
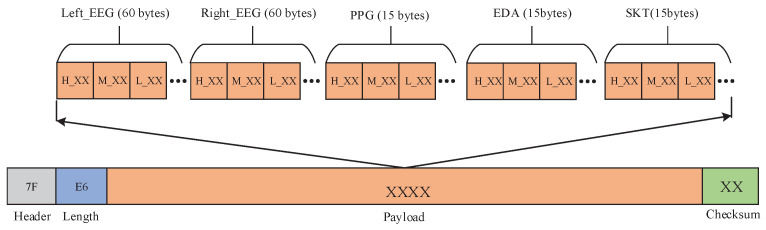
The data packet format.

Data visualization interface is mainly used to complete the process of the real-time waveform display of multimodal bio-signals, the communication rate and port number setting, and the data storage. The visualization interface program design mainly includes two threads. One is responsible for data receiving, unpacking, verification, etc, and the other is mainly responsible for waveform drawing. Data receiving module receives bio-signals data from MCU through Bluetooth according to certain data packet format. The data packet format is shown in Figure 8. In this paper, the sampling frequency of EEG is 400 samples per second. Since other peripheral bio-signals are low frequency signal, they are recorded at a sampling frequency of 100 Hz. The data packet consists of 168 bytes including a header byte (0x7F), a payload length byte (0xE6), data payload packet and a CRC checksum byte. After calculation, 20 data packets of 168 bytes each in size need to be transmitted per second. Every 50 ms, a data packet is transmitted to PC. Therefore, the establishment connection time is 50 ms. Bluetooth is in sleep state when there is no data transmission, which can reduce the number of data transmission, thereby reducing the power consumption of Bluetooth.

**Figure 9:**
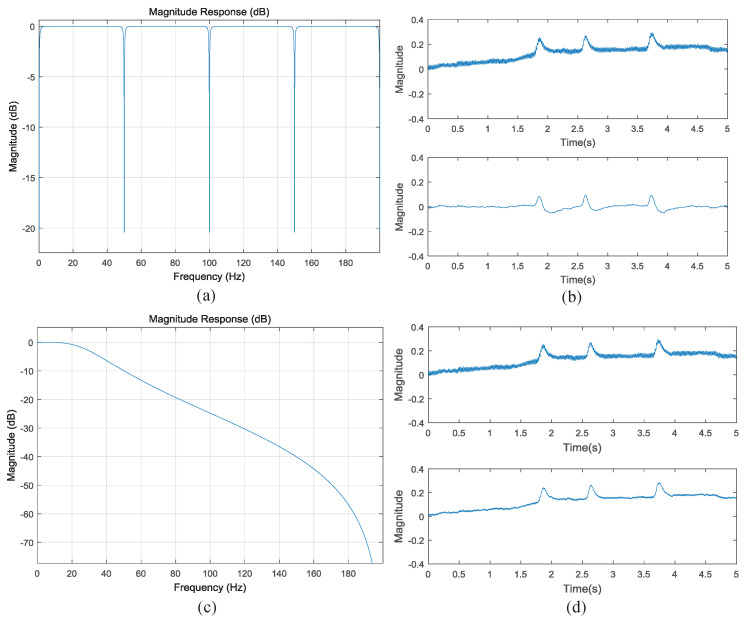
Response of the filter and examples of the filter effect: (**a**) the frequency response of the comb filter. (**b**) an example of blinking EEG raw data and the output EEG data with the comb filter. (**c**) the frequency response of the low-pass filter. (**d**) an example of blinking EEG raw data and the output EEG data with low-pass filter.

After receiving one data packet sent by BLE, the PC unpacks and verifies it according to the data format shown in Figure 8. By observing the time-domain and frequency-domain waveform of the two-channel EEG signals, it can be found that there is partial power line interference in the collected data. Considering the low energy and small size of wearable devices, we did not design an analog 50-Hz notch filter when designing the AFE circuit of EEG. Therefore, a digital comb filter has been implemented on the PC to eliminate power line interference. In this paper, the Filter Design & Analysis Tool (FDATool) in MATLAB (Mathworks, Natick, MA, USA) signal processing toolbox is used to design filter. The FDATool interface provides an interactive design environment for filter design. We choose the infinite impulse response (IIR) comb filter with eighth-order and 1-Hz bandwidth. From the frequency response of the comb filter in Figure 9a, it can be seen that there is an attenuation of 20.4 dB at 50 Hz and its frequency doubling. The difference equation of the comb filter is as follows:

(12) $$y\left(n\right)=\frac{1+rh0}{2}\left[x\left(n\right)-x\left(n-N\right)\right]+rh0\left[y\left(n-N\right)\right],$$

where *N* = 8 is the order of the filter. x(n) and y(n) represent input and output signal, respectively. rh0 represents filter coefficient which is generated by FDATool with the value of 0.96852105385218623. The blinking EEG data collected from the forehead before and after passing through the comb filter are shown in Figure 9b. It can be seen that the filtered EEG signal attenuates 50-Hz power line interference.

The frequency of emotion-related EEG signal ranges from 0.5 to 30 Hz, and after the comb filter processing, there may still be some high frequency interference greater than 30 Hz. Therefore, a low-pass filter after the output of the comb filter is applied to further eliminate the high frequency interference. In this paper, a direct II type Butterworth IIR low-pass filter with a cut-off frequency of 30-Hz is designed. Considering the real-time display of EEG signal, the filter order is set to be 2. The forward and feedback channels are respectively expressed as:

(13) $$w\left(n\right)=x\left(n\right)-\sum_{i=1}^{2}a_{i}w\left(n-i\right),$$

(14) $$y\left(n\right)=x\left(n\right)-\sum_{j=0}^{2}b_{j}w\left(n-j\right),$$

where $a_{i}$ and $b_{j}$ are filter coefficients. The feedback value w(n) after iteration can be calculated by substituting the input signal x(n) into Equation (13), then the output signal y(n) can be calculated according to Equation (14). Figure 9c represents the frequency response of the second-order low-pass filter, and Figure 9d describes the waveform before and after the blinking EEG data passing through the low-pass filter.

Since other peripheral bio-signals are known to be low frequency signals, different cuff-out frequency low-pass filters are designed to meet the requirements according to the above method. The filtered data can be drawn in real-time by calling the drawing thread. Data visualization interface can display the real-time SKT and PR value. It can also save the current collected data to the local document at any time.

### 4.2. Experimental Paradigm Setting Interface

The experimental paradigm interface is used to set the experimental paradigm according to the requirements of researchers. It mainly includes the following functions.

(1) VR scene selection: Users can select the VR scene required for this experiment from the VR scene library;
(2) Parameter setting: Users can set the length parameters of subjects immersed in VR environments, including prompt time, VR scene playback time and questionnaire survey time, etc;
(3) VR scene play: Users start the VR scene by clicking the ‘Play’ button. When the experiment time reaches the specified value, the VR scene automatically stops playing. The ‘Stop’ button and the ‘Reset’ button are used to stop playing the VR scene and reset the parameters, respectively;
(4) Interactive control: Users interact with the controls on the interface to change the parameters, so as to send instructions to the system and finally realize the function.

### 4.3. Emotion Modeling Interface

Emotion modeling interface mainly includes data pre-processing, feature extraction, feature dimension reduction, feature standardization, model training, model preservation, model loading, emotion classification, etc.

(1) Data Pre-processing: Using digital IIR filter such as Butterworth type, Chebyshev I type. According to the requirements, user can choose low-pass, high-pass, band-pass, band-stop four kinds of filters and then set the sampling frequency, order, cut-off frequency and other parameters;
(2) Feature Extraction: Extracting time-domain, frequency-domain, time-frequency domain and nonlinear feature extraction. User can choose multi-feature fusion function of multimodal bio-signals;
(3) Feature Dimensionality Reduction: Using different feature dimensionality reduction methods to avoid “dimension disaster”, including principal component analysis (PCA) and linear discriminant analysis (LDA). User can set the number of dimensions required for dimension reduction;
(4) Feature Standardization: Including Z-score standardization, maximum and minimum value standardization;
(5) Feature Classification: Selecting common classifier and set parameters. The common classifiers are Support Vector Machine (SVM), Logistic Regression (LR), Random Forest (RF), Bayesian Network (BN) and Decision Tree (DT).

**Figure 10:**
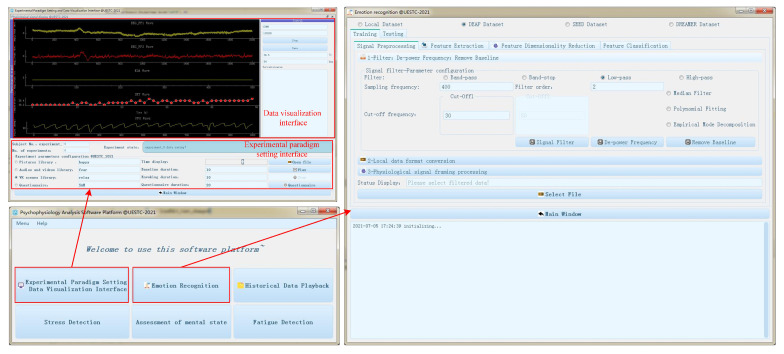
The schematic diagram of the HCI interface.

The schematic diagram of the HCI interface is shown in Figure 10.

## 5. Performance Evaluation

### 5.1. System Structure Evaluation

We tested the performances of portability, comfort and ease of wearing of the HMD Bio Pad system and compared with those of Biopac, Mindwave and E4 watch. Eight healthy individuals were recruited as volunteers to evaluate the level of portability, comfort and ease of wearing. We used a scale of 1–5, where 1 is minimum level and 5 is maximum level. Table 1 gives a summary of the average level of portability, comfort and ease of wearing by participants of each device. It can be seen that the average level of HMD Bio Pad in portability, comfort and ease of wearing are better than that of Biopac or Mindwave. It can be observed that the comfort level of E4 watch is sightly higher than that of HMD Bio Pad. However, HMD Bio Pad can collect EEG signals.

### 5.2. EEG Acquisition System Evaluation

In order to evaluate the performance of EEG acquisition system, two common experiments are provided in the following parts. Five healthy volunteers were recruited to evaluation the EEG acquisition system.

#### 5.2.1. EEG Eyes-Blink Task

**Figure 11:**
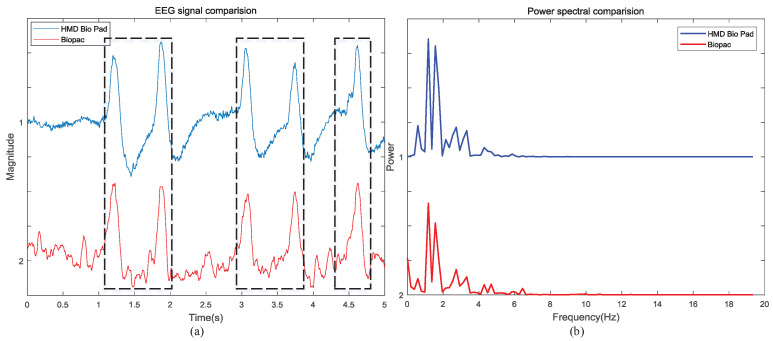
An example of eyes-blink task: (**a**) An example of FP1 region EEG signal obtained by HMD Bio Pad and Biopac. (**b**) EEG power spectral density of (**a**).

In this paper, EEG signals in the FP1 area are recorded simultaneously by HMD Bio Pad and the EEG module of Biopac, USA. The correlation between the EEG signal collected by the two devices with mental dry electrodes is observed after normalization. In Figure 11a, the pulse signal encircles by black dotted line corresponds to the eyes blink, which indicates that the EEG signals collected by the two devices have a strong consistency and prove the effectiveness of HMD Bio Pad. Meanwhile, the power spectral density also indicates that the two systems have the same spectral components in Figure 11b. The Pearson correlation coefficient between the power spectral density of both EEG signal is 0.945, which indicates a strong linear positive correlation between these two signals. The above results demonstrate that the proposed EEG acquisition system can achieve the comparable signal quality in comparison with the dedicated bio-signals measuring device. While in FP1 area, although the recorded EEG signal has high similarity, most of EEG signal is drowned by eyes blink. This may not be very persuasive for EEG measurement. To further verify the performance of HMD Bio Pad in EEG measurement, an eyes-open and eyes-closed task was performed.

#### 5.2.2. Eyes-Open and Eyes-Closed Task

A common method used to verify whether EEG signal can be collected correctly is the eyes-open and eyes-close task [32]. Quantities studies have revealed that when subjects are in wakeful relaxation with eyes closed, clear alpha rhythm can be observed generally in the frequency range of 8 to 13 Hz. When the subjects open their eyes, the alpha rhythm reduces. In order to verify the performance of EEG acquisition system, we performed an eyes-open and eyes-close task. In this task, the subjects were lying comfortably in a room with dim light and less sound interference, and the HMD Bio Pad was used to acquire EEG signal in the FP1 area. The EEG data consists of 30 s wakeful relaxation with eyes closed data and 30 s of eyes open data. Excluding the influence of psychological factors and eyes blink in the experiment, the clean data of 15 s in eyes open and eyes closed data were selected, respectively.

**Figure 12:**
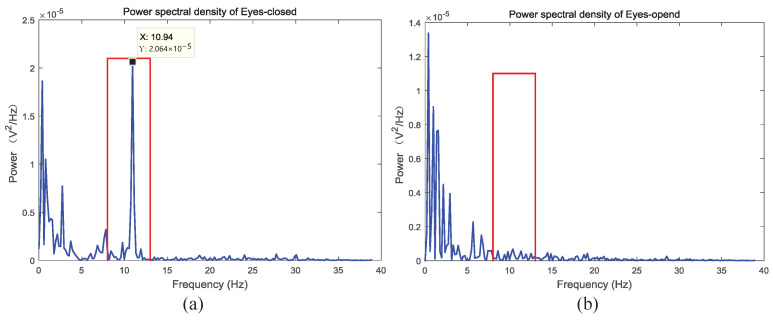
An example of eyes-open and eyes-closed task: (**a**) EEG power spectral density of eyes-open. (**b**) EEG power spectral density of eyes-closed.

Figure 12 demonstrates the EEG power spectral density of eyes-open and eyes-closed task. It can be seen from the figure that compared to the eyes open state, the dominant frequencies of the EEG power spectral density is in the frequency range of 8 to 13 Hz when the subject are in eyes closed state. It proved that the proposed system can efficiently record the EEG signal in two different states.

### 5.3. EDA Acquisition System Evaluation

#### 5.3.1. Signal-to-Noise Ratio

SNR represents the ratio, in dB, between signal and noise. For the SNR of EDA signal, we refers the method mentioned in reference [28]. The method uses the sum of power spectral density in the range of (0, 5] Hz as the useful signal, and the signal beyond frequency range is the noise signal. The SNR of EDA can be calculated by Equation (15):

(15) $$SNR=\frac{PSD_{EDA(0,5]Hz}}{PSD_{EDA(5,Fs/2]Hz}},$$

where Fs represents the sampling frequency. Referring to the experimental paradigm of EDA signal acquisition in reference [28], 5 subjects were required to use HMD Bio Pad to collect data for 2 min and 30 s (4 relaxation phases of 30 s and 3 hyperventilation phases of 10 s) in a quiet room with temperature of 25 ${}^{{}^{\circ}}C$. The data were divided into 29 segments according to a segment of 10 s (50% overlap), and the SNR of each segment was calculated respectively. By comparing the calculated mean SNR with the commercial device and open source hardware mentioned in reference [28], it can be seen from Table 2 that the average SNR of EDA signal recorded in this paper is 28.52 dB under the similar sampling frequency. In comparison to other commercial devices or open source hardware, the EDA acquisition system achieves similar SNR and the minimum standard deviation (0.41 dB), which indicats that the SNR of EDA in this paper is relatively stable. SNR can not fully prove the effectiveness of EDA signal, this paper further verifies the performance of EDA acquisition system using skin conductance reaction (SCR).

#### 5.3.2. Skin Conductance Reaction

**Figure 13:**
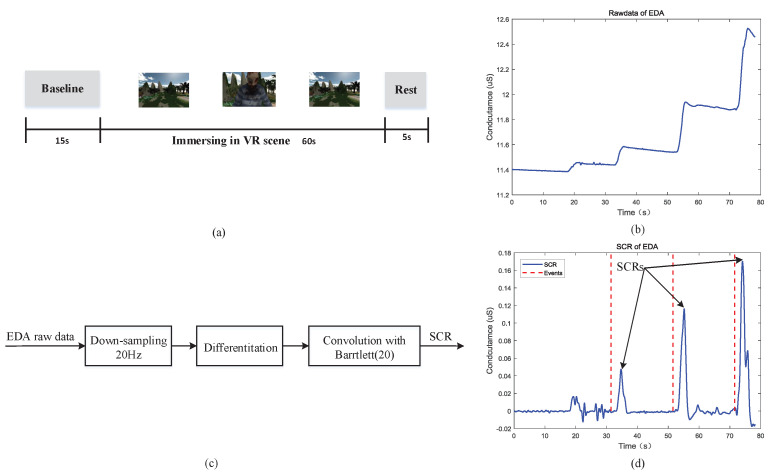
An example of SCR extraction: (**a**) Experimental paradigm of EDA acquisition. (**b**) An example of raw data of EDA under emotional stimulation. (**c**) Block diagram of SCR detection module. (**d**) SCR extraction from (**b**) using (**c**) method.

The skin conductance response (SCR) in EDA signal is considered to be a vital feature because it responds to the internal response of the human body to external stimulus. In this paper, the virtual scene is used as the stimulus source to observe the SCR after stimulation for verifying the performance of the EDA acquisition system. According to the instructions of the experiment instructor, the subjects firstly wore HMD Bio Pad and then recorded baseline EDA data for 20 s in a quiet room with temperature of 25 ${}^{{}^{\circ}}C$. Then subjects were immersed in the virtual village scene for 60 s. During the process of scene playing, stimulus events would appear regularly in the scene. The experimental paradigm and scene are shown in Figure 13a. The EDA signal recorded in the experiment is shown in Figure 13b. In this paper, the SCR extraction method mentioned in literature [33,34] is used to extract SCR from the EDA signal. The process of calculating SCR is shown in Figure 13c. The EDA raw data is first sampled down to 20 Hz, then differentiated, and the differentiated signal is convolved with the 20 points Barlett window. The SCR signal is shown in Figure 13d, and the red dotted line represents the moment when the triggering event occurs. It can be seen from the figure that the SCR occurs after a few seconds of the stimulus event, which is consistent with the conditions of SCR generation described in literature [34]. This indicates that the EDA acquisition system can correctly collect EDA signal and extract SCR signal related to stimulus response from EDA signal, which verifies the feasibility of the EDA acquisition system.

### 5.4. SKT Acquisition System Evaluation

**Figure 14:**
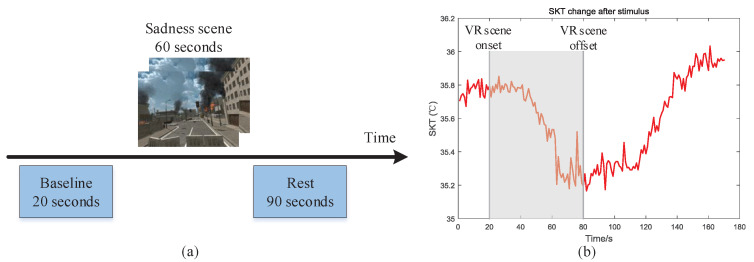
An example of SKT acquisition under emotional stimulation: (**a**) Experimental paradigm of SKT acquisition. (**b**) An example of SKT under emotional stimulation.

As mentioned above, we use a contact temperature sensor (LMT70) for skin temperature (SKT) acquisition. Numerous studies have showed that unpleasant emotions like sadness, fear, anxiety may cause the decreasing of SKT temperature [1,35]. In order to validate the performance of SKT acquisition system, we designed a experimental to observe SKT of subjects who immersed in the sadness VR scene. The experimental paradigm is designed according to the literature [1] and showed in Figure 14a. The subjects firstly wore HMD Bio Pad and then waited a few seconds until the temperature stabilized in a quiet room with room temperature of 25 ${}^{{}^{\circ}}C$. Next, we recorded 20 s baseline data, 60 s immersing in sadness VR scene data, followed by 90 s resting data allowing the thermal response to build up.

The collected SKT data are smoothed using a three point moving average algorithm with 3 weights (0.8, 0.1, 0.1). A SKT response example when the subject immersed in the sadness ruin scene is shown in Figure 14b. It can be seen that the SKT of the subject firstly keep stable in the baseline. Then the SKT decreases about 0.2 ${}^{{}^{\circ}}C$ between the beginning and the end of stimulation. Finally, the SKT rises after the stimulations. The SKT change curve is consistent with the conclusion that SKT decreases in unpleasant scenes in literature [1]. Therefore, the proposed SKT acquisition system can correctly collect the SKT changes in the negative emotional scenes, which verifies the feasibility of the system.

### 5.5. PPG Acquisition System Evaluation

Pulse rate (PR) is an important reference feature in the research of emotion recognition. In view of the random swing of the head and movement of the body in the virtual environments, the light path of the PPG sensor illuminating the skin will change irregularly, leading to light leakage phenomenon. Therefore, the reflected light intensity contains random interference, which makes the collected PPG signal contain motion artifact (MA). In this paper, the method proposed in literature [36], which used the parallel RLS adaptive filtering algorithm with acceleration signal as reference signal is adopted to attenuate MA. Then the spectral peak tracking with verification based on the FFT method is used to estimate PR value. Eight heart-healthy subjects were immersed in VR scenes of joy, calmness, sadness and fear for 60 s. Each scene was separated by 30 s (relaxation music), and a total of 6 min of data were collected. The two-channel PPG signals, tri-axis acceleration signals and one-channel ECG signal using wet electrodes firmly attached on the chest were recorded simultaneously by HMD Bio Pad (reserved ECG analog channel). The function of the one-channel ECG signal was applied to extract the heart rate (HR) value, which was used as ground-truth value. We estimated PR in 8 s for each time window. There are 6 s overlaps between the successive time windows. After the calculation, about 180 windows of PR could be recorded in each recording. In this paper, we evaluate the performance of our method using the average absolute error (AAE), Bland-Altman plot and Scatter plot. The mean average absolute error (AAE) is defined as

(16) $$AAE=\frac{1}{W}\sum_{i=1}^{W}\left|PR_{est}\left(i\right)-PR_{true}\left(i\right)\right|,$$

where *W* represents the number of time windows and $PR_{est}\left(i\right)$ represents the estimated PR in the *i*-th time window using our proposed method. The ground-truth PR $PR_{true}$ is extracted from the simultaneous ECG signal in each time window.

**Figure 15:**
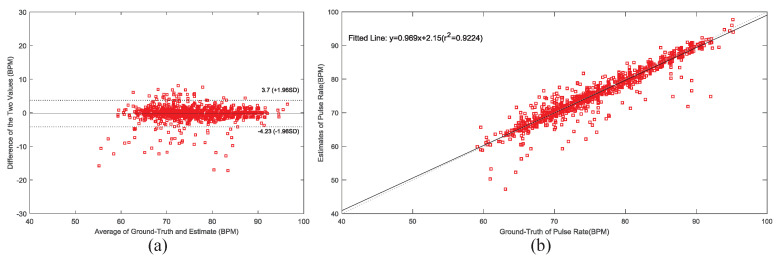
The Bland-Altman plot and Scatter plot: (**a**) Bland-Altman plot over 8 recordings. (**b**) Scatter plot between the ground-truth and the estimated PR values over 8 recordings.

Table 3 shows the mean AAE of PR for each subject using the parallel RLS algorithm calculation. As can be seen from the Table 3, the mean AAE of the 8 recordings is 1.12 ± 1.53. Figure 15a shows the Bland-Altman plot, the limit of agreement (LOA) is [−4.23,3.70] BPM which shows that 95% data exist within 1.96σ from mean. Figure 15b shows the Pearson correlation plot over 8 recordings. The Pearson correlation is 0.960. It can be seen from the above results that the PR value can be accurately estimated from the collected forehead PPG signal by PPG acquisition system with the method of literature [36].

### 5.6. Software

The application of the HCI interface, including data visualization interface, the emotional experiment paradigm setting, and the emotion recognition modeling interface have been tested and evaluated. The real-time bio-signals in the data visualization interface can be correctly displayed and stored in the local file. The emotion experimental paradigm setting can correctly play the experimental scene, set the experimental scene duration and interval time parameters after testing. The emotion recognition modeling interface can easily perform pre-processing, feature extraction, feature dimension reduction and feature classification for multimodal bio-signals. The model after training can also be correctly imported into the real-time emotion recognition system to determine the current emotional state.

## 6. Conclusions

In this paper, a wearable multimodal bio-signals acquisition system called HMD Bio Pad is developed. The HMD Bio Pad is connected to the VR HMD using a velcro fastener, and the multimodal bio-signals acquired from the forehead are transmitted to the HCI interface via BLE. Metal dry electrodes were used to record EEG signals in FP1 and FP2 area. The consistency of EEG signal with eyes blink between HMD Bio Pad and Biopac is verified by eyes-blink task. The eyes-open and eyes-closed task indicates that the power spectral density of alpha rhythm in the frequency range of 8 to 13 Hz with eyes closed state is much higher than that with eyes open state. The above two tasks show that the EEG acquisition system designed in this paper is feasible. The feasibility of EDA acquisition system is proved from the SNR and the SCR extracted from EDA signal after stimulation. The SKT acquisition system can monitor the SKT changes of subjects in the unpleasant scene. Real-time PR value is estimated from the collected forehead PPG signal. According to the results, the mean AAE of PR is 1.12 ± 1.53 among 8 recordings. The above results show that the proposed HMD Bio Pad can effectively record multimodal bio-signals. At the same time, the functions of HCI interface software are tested, and the results indicate that the software can correctly perform all functions. In future work, we will incorporate more bio-signals related to emotion recognition into HMD Bio Pad such as EMG, electrooculography (EOG) and oxyhemoglobin saturation (SpO2).

## Figures and Tables

**Table 1:** Comparison of Portability, Comfort and Ease of wearing of different devices.

Device	Portability	Comfort	Ease of Wearing
HMD Bio Pad	4.6	4.4	4.7
Biopac	1.4	2.5	1.8
Mindwave	3.6	2.6	3.0
E4 watch	4.3	4.8	4.0

**Table 2:** Mean and standard deviation of SNR for EDA signal.

Device	HMD Bio Pad	PSD-D	EH	QS	ML
Mean_SNR (dB)	28.52	29.83	27.25	19.82	34.18
SD_SNR (dB)	0.41	0.68	2.46	6.96	10.25

**Table 3:** AAE in BPM used algorithm structure in [36].

Recording #	1	2	3	4	5	6	7	8	μAAE±SD
Parallel RLS [36]	1.75	1.31	1.01	1.15	1.20	0.85	0.57	1.10	1.12 ± 1.53

## Data Availability

The data presented in this study are available on request from the corresponding author.
